# Hidden systems in primary care cancer detection: an embedded qualitative intervention development study

**DOI:** 10.3399/BJGP.2023.0339

**Published:** 2024-07-09

**Authors:** Julia Hiscock, Rebecca-Jane Law, Kate Brain, Stephanie Smits, Sadia Nafees, Nefyn H Williams, Jan Rose, Ruth Lewis, Jessica L Roberts, Annie Hendry, Richard D Neal, Clare Wilkinson

**Affiliations:** North Wales Centre for Primary Care Research (NWCPCR), Bangor University, Wrexham.; North Wales Centre for Primary Care Research (NWCPCR), Bangor University, Wrexham.; Division of Population Medicine, School of Medicine, Cardiff University, Cardiff.; Division of Population Medicine, School of Medicine, Cardiff University, Cardiff.; North Wales Centre for Primary Care Research (NWCPCR), Bangor University, Wrexham.; Department of Primary Care and Mental Health, Institute of Population Health, University of Liverpool, Liverpool.; North Wales Centre for Primary Care Research (NWCPCR), Bangor University, Wrexham.; North Wales Centre for Primary Care Research (NWCPCR), Bangor University, Wrexham.; North Wales Centre for Primary Care Research (NWCPCR), Bangor University, Wrexham.; North Wales Centre for Primary Care Research (NWCPCR), Bangor University, Wrexham.; DISCO (Diagnosis of Symptomatic Cancer Optimally), University of Exeter, Exeter.; North Wales Centre for Primary Care Research (NWCPCR), Bangor University, Wrexham.

**Keywords:** general practice, early diagnosis, delayed diagnosis, decision making, Wales, cancer

## Abstract

**Background:**

UK cancer mortality is worse than in many other high-income countries, partly because of diagnostic delays in primary care.

**Aim:**

To understand beliefs and behaviours of GPs, and systems of general practice teams, to inform the Think Cancer! intervention development.

**Design and setting:**

An embedded qualitative study guided by behaviour change models (COM-B [Capability, Opportunity, Motivation – Behaviour] and theoretical domains framework [TDF]) in primary care in Wales, UK.

**Method:**

Twenty qualitative, semi-structured telephone interviews with GPs were undertaken and four face-to-face focus groups held with practice teams. Framework analysis was used and results were mapped to multiple, overlapping components of COM-B and TDF.

**Results:**

Three themes illustrate complex, multilevel referral considerations facing GPs and practice teams; external influences and constraints; and the role of practice systems and culture. Tensions emerged between individual considerations of GPs (Capability and Motivation) and context-dependent external pressures (Opportunity). Detecting cancer was guided not only by external requirements, but also by motivational factors GPs described as part of their cancer diagnostics process. External influences on the diagnosis process often resulted from the primary–secondary care interface and social pressures. GPs adapted their behaviour to deal with this disconnect. Positive practice culture and supportive practice-based systems ameliorated these tensions and complexity.

**Conclusion:**

By exploring individual GP behaviours together with practice systems and culture we contribute new understanding about how cancer diagnosis operates in primary care and how delays can be improved. We highlight commonly overlooked dynamics and tensions that are experienced by GPs as a tension between individual decision making (Capability and Motivation) and external considerations, such as pressures in secondary care (Opportunity).

## Introduction

The importance of timely and early-stage diagnosis of cancer is well established.^1^ Cancer survival rates are low in the UK (England 5-year relative survival 47% in men and 53% in women) compared with the European average (50% for men and 58% for women)^2^ highlighting the potential for initiatives, including those aimed at increasing the proportion of early-stage diagnoses,^3^ to improve survival in the UK. At least 60% of people with cancers present with symptoms to primary care.^4^^,^^5^ Additionally, many patients who are diagnosed through other routes (including ‘emergency diagnoses’) initially present with symptoms to primary care.^5^ Hence, GPs, as the first point of contact in a free at the point of use system, have a pivotal role in assessing symptoms that may be cancer, the selection of patients for referral (whether urgent or routine), and diagnostic investigations.

Criteria for referral for suspected cancer in the UK is determined by the National Institute for Health and Care Excellence (NICE) NG12 and is based on specific combinations of symptoms, signs, and test results, whether alone or in combination.^6^ However, there is significant variation in UK practice^7^^–^9 and almost half of avoidable delays in cancer diagnosis occur within primary care.^10^ The reasons for this are poorly understood and are likely to be multifactorial but may include differences in clinical decision making and referral strategies used by individual GPs,^11^ ambiguity in NICE guidance,^12^^,^^13^ and access to diagnostic testing.^14^ Furthermore, little is known about influences on the cancer referral process at the level of individual GPs and the systems in which they operate.^15^ Understanding this granular detail about GP approaches, attitudes, behaviours, and experiences is crucial to ensuring that initiatives to improve the early diagnosis of cancer are effective.

**Figure 1.**
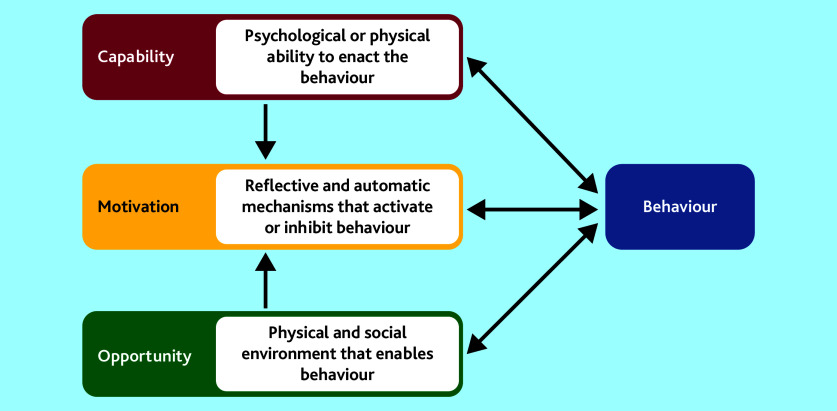
COM-B model for understanding behaviour of the behaviour change wheel. Reproduced from Holloway *et al*, 2020,^17^ under Creative Commons Attribution Non Commercial (CC BY-NC 4.0) open access licence.

The aim of this qualitative study reported here was to understand the beliefs and behaviours of UK NHS GPs and the systems of practice teams in the diagnosis of cancer to inform the ThinkCancer! intervention development. The research used qualitative methods and was guided by the COM-B Model, which is a theoretical framework that can be used for understanding behaviour.^16^ Capability (C), Opportunity (O,) and Motivation (M) are components needed in order for Behaviour (B) to be changed. The model states that Capability can be psychological or physical, Opportunity can be social or physical, and Motivation can be automatic or reflective (see Figure 1).^16^

**Table:** How this fits in

Delays in primary care contribute to high cancer mortality in the UK. Multiple tensions in cancer detection and referral occur at individual, practice, and primary–secondary interface levels. The rejection, or ‘downgrading’, of GP referrals led to frustration and complex workarounds. Positive practice cultures and systems can ameliorate tensions, reinforcing the importance of whole-practice interventions.

## Method

This qualitative study was guided by the COM-B model and the theoretical domains framework (TDF). The work presented here was embedded within a comprehensive, mixed-methods programme of research and intervention development for the ThinkCancer! trial of a multicomponent primary care educational intervention designed to increase knowledge and awareness of potential cancer symptoms and to ultimately lower referral thresholds and improve cancer outcomes.^18^ The intervention incorporates a whole-practice approach that is designed to improve and revive general practice systems that facilitate communication about cancer and, in turn, improve referral and detection.^18^

### COM-B model and TDF

The COM-B model^16^ and TDF^19^ describe how changing Behaviour is a result of changing one or more components of psychological and/or physical Capability (knowledge, skills, and abilities to enact the Behaviour), social and physical Opportunity (external factors that enable the Behaviour to occur), and automatic and reflective Motivation (internal processes that influence decisions and behaviours) (see Figure 1).^16^

The TDF provides a more granular lens through which to understand Capability (physical skills; knowledge; cognitive and interpersonal skills; memory, attention, and decision processes; and behavioural regulation), Opportunity (social influences; and environmental context and resources), and Motivation (reinforcement; emotions; social/professional role and identity; beliefs about capabilities; beliefs about consequences; and goals and intentions).^19^ The COM-B model and TDF can be used together to identify what needs to change to bring about the target behaviour. The application of COM-B and TDF occurred at each stage of the study.

### Participant selection

#### Sampling

This study was carried out in Wales between 2016 and 2019 when there were approximately 2000 GPs and 450 practices. Purposive sampling, from publicly available websites and databases, of individual GPs and practices enabled appropriate inclusion of a range of relevant characteristics.

#### One-to-one telephone interviews with GPs

The purposive sampling criteria for individual GPs were practice rurality, deprivation level, and years since first medical qualification. To enable the inclusion of a range of experiences and influences, we monitored sample characteristics such as whether the GPs were locums, salaried, partnered or registrars, as well as their gender.

#### Practice-team focus groups

Four practices were purposively sampled by practice characteristics (training practice status and practice rurality), area-level deprivation,^20^ and region. To facilitate attendance of sufficient participants and enable discussion on general practice cultures, systems, and norms, we selected practices with a minimum size of 5000 patients. We aimed to include 6–8 participants (maximum of 10) per focus group. The focus groups were whole-team groups and included GPs, practice nurses, practice managers, practice receptionists, and administrative team members.

### Recruitment

#### One-to-one telephone interviews with GPs

GPs were invited to take part by the second author through email and follow-up telephone calls. A sample log was developed to record fit with the purposive sampling criteria.

#### Practice-team focus groups

Practices received an initial email with an invitation to participate letter signed by the senior author. The focus groups practices were then recruited by telephone calls and one follow-up call by the second author to the practice managers, according to the sampling frame. Information on reasons for refusal relating to recruiting participants to interviews and focus groups were not collected.

### Data collection

The data came from two separate sources: one-to-one telephone interviews with GPs and practice-team focus groups.

#### One-to-one telephone interviews with GPs

Telephone interviews explored the underlying beliefs and behaviours of individual GPs in identifying, investigating, and referring cancer signs and symptoms. A semi-structured topic guide was co-produced with input from patient and public involvement (PPI) and the project team, including study GPs and those with COM-B expertise (the first seven authors, the ninth author, the eleventh author, and the senior author). The topic guide was piloted and aligned to the COM-B model (see Figure 1) and TDF.

One pilot interview with a GP known to the research team was conducted to test the functionality of the topic guide. At the end of the interview, demographic information was collected to assist with analysis and reporting. Interviews used probes and open-ended questioning to obtain in-depth accounts. They were audio-recorded (with permission). Interviews were conducted by the second author, a non-clinical health services researcher with qualitative research experience. Participating GPs were offered a £30 shopping voucher, with an additional £30 charity donation.

#### Practice-team focus groups

Focus groups were conducted at a time convenient for the practice. The discussion was based on a co-produced topic guide (as for the GP interviews above), with prompts and probes that further explored findings from the interviews and other elements of the Think Cancer! programme. Background and demographic information were collected at the end of the focus group to assist with analysis and reporting. The groups were audio-recorded (with permission) and anonymised. The focus groups were conducted face-to-face by the first and second authors, who are non-clinical health services researchers with qualitative research expertise. Participating practices were reimbursed £250 for their involvement. Ethical approval was obtained for both the interviews and focus groups as part of the overall programme of research.

### Data analysis

Both datasets were transcribed verbatim by an independent transcriber, checked, corrected, and anonymised by the interviewer (the second author). Data were analysed using framework analysis,^21^ a matrix-based analysis. All five stages of framework analysis were followed: familiarisation, thematic framework identification (developing the coding tree, or ‘index’), indexing (coding), charting, mapping, and interpretation.

Themes were derived from the data to produce the ‘index’ (coding tree). Microsoft Excel was used to organise and synthesise the data into the matrix. A descriptive analysis of the themes was conducted, followed by interpretative analysis. This more in-depth, explanatory level of analysis was conducted using the charts. It involved identifying patterns, links, and associations within the data. The interviewer (the second author), conducted all stages of the analysis, supported by the senior qualitative colleague (the first author). In addition, the full team were involved in interpretative analysis sessions. These were held with the wider project team, including those contributing COM-B/TDF expertise. The purpose of these was to map, discuss, scrutinise, and, eventually, finalise the emerging themes.

### Research team and reflexivity

Telephone interviews were conducted by the second author, an experienced researcher who was supervised by a senior qualitative researcher (the first author), both have PhDs and are university non-clinical health services researchers. Focus groups were conducted by the first and second authors. Neither the first or second authors had established relationships with any of the participants before study commencement. At the outset of the interviews and focus groups the second author explained their background as a non-clinical researcher and informed participants of the study purpose and funder.

## Results

Twenty GPs, with representation from both urban and rural settings and varying levels of socioeconomic deprivation, were recruited. The interviews lasted approximately 30–45 min and the GPs were from mostly separate practices across Wales. Focus groups were conducted with four practices covering North and South Wales that lasted approximately 1 h. The size of the focus groups ranged from 4–11 participants. Focus group participants occupied a range of roles within the practice team, although GPs outnumbered other professional groups.

The three main themes reported here illustrate the complex, multilevel considerations facing GPs and practice teams in the process of cancer referral. This begins with a description of a range of GPs’ personal considerations, attitudes, and behaviour in cancer diagnosis and referral. This is then combined with an account of the external influences and constraints that affect the process. Finally, the data on the practice systems and culture are described, adding how these can be helpful dealing with the tensions described. Subthemes were mapped on to multiple, overlapping components of the COM-B model and TDF, as shown in Box 1.

**Box 1.** Qualitative results mapped to COM-B^a^ and theoretical domains framework (TDF)

**Qualitative subtheme**	**COM-B factor**	**TDF domain**
Personal standards and integrity	**Reflective Motivation** Physical **O**pportunity (environmental context and resources)	• Social/professional role and identity
Personal emotions		Beliefs about capabilities Beliefs about consequences
Relationships and reputation		• Social/professional role and identity
Autonomy and ‘gut feeling’	Automatic **M**otivation (emotion) Social **O**pportunity (social influences)	• Beliefs about capabilities
Guidelines	**Psychological Capability** Reflective **M**otivation (beliefs about capabilities)	• Memory, attention, and decision processes
Downgrading or rejection of referrals	**Physical Opportunity** Social **O**pportunity (social influences) Automatic **M**otivation (emotion) Psychological **C**apability (memory, attention, and decision processes) Reflective **M**otivation (social/professional role and identity)	• Environmental context and resources
Adaptations	**Social Opportunity** Physical **O**pportunity (environmental context and resources) Psychological **C**apability (cognitive and interpersonal skills) Reflective **M**otivation (social/professional role and identity)	• Social influences
Practice culture	• Physical **O**pportunity (environmental context and resources)	• Social influences
Practice systems	• Social **O**pportunity (social influences)	• Environmental context and resources

a *Capability (C), Opportunity (O) and Motivation (M) are the components needed in order for Behaviour (B).*

### Personal considerations and sources of influence

The work of cancer diagnosis and referral for GPs was personal as well as professional and clinical. We identified four main personal considerations that GPs described as part of their cancer diagnostics processes, reflecting Motivation — personal standards and integrity, personal emotions, relationships and reputation, and autonomy and ‘gut feeling’ (Box 1).

#### Personal standards and integrity

GPs talked about the importance of ‘doing a proper job’, with some describing a need to be ‘perfect’ in line with their professional role and identity (reflective Motivation). Yet GPs also reported feeling frustrated about their ability to meet the standards to which they aspired at multiple points within the diagnostic and referral process. This overlapped with concerns about the consultation time being too short for adequate diagnostic or risk assessment activities, reflecting physical Opportunity (environmental context and resources).

#### Personal emotions

Salient factors for GPs in the process of considering urgent suspected cancer referral processes included personal feelings about beliefs in their capabilities. These included occasionally wavering confidence, and a fear of making mistakes and the consequences of missing a potential cancer (reflective Motivation).

#### Relationships and reputation

GPs reported that maintaining the doctor– patient relationship in cancer diagnosis was important, both personally and to their professional identity (reflective Motivation). They described how it was important to them to inspire confidence in their patients and not show weakness — despite sometimes feeling a lack of self-assurance.

#### Autonomy and ‘gut feeling’

Some GPs reported that a sense of autonomy within the diagnostic process was important. They felt that prescriptive and guideline-driven requirements acted against some of the strengths they felt they brought to the diagnostic process (reflective Motivation). Automatic Motivation was evident, for example, many GPs emphasised the importance of following their intuition or ‘gut feelings’. This was easier when they knew the patient and could tell if the patient seemed out of character. However, it was made more difficult by external expectations, including social norms and pressures to comply with guidelines that they felt demanded a different approach (social Opportunity):

*‘ When I have a gut feeling I follow it through and I say, “No, I think we should do something”.’*

(Rural, low deprivation, >30 years since first medical qualification)

### External influences

The process of detecting cancer at the GP level was guided not only by GPs’ personal considerations, but also by a range of internal and external requirements, influences, and pressures reflecting Capability, Motivation, and Opportunity. These influences included clinical guidelines, and second, the dynamic between primary and secondary care, in particular the ‘downgrading’ of GP urgent referrals (Box 1).

#### Guidelines

GPs discussed the role played by NICE and other guidelines in their referral decisions. For most, the guidelines performed several useful functions, primarily in supporting the cognitive processes surrounding their clinical decision making (psychological Capability). Some described tensions between adherence to guidelines and following the personal approaches described above. Guidelines performed both clinical and emotional functions for GPs, by reducing the cognitive load and emotional burden associated with clinical decision making and providing reassurance that a decision was made systematically (reflective Motivation). This reduction in cognitive and emotional burden could sometimes curtail personal autonomy and ‘gut feeling’. By describing the NICE guidelines as ‘comforting’ this GP’s account refers us back to the personal feelings and emotions discussed in the previous section and demonstrates how guidelines can support reflective Motivation:

*‘I find them* [the guidelines] *quite comforting, because they give me a framework … you need something to hang your decision on, so if it’s a wrong decision at least I can say, “it’s a wrong decision, but I based it on that”.’*

(Urban, high deprivation, 15–25 years since first medical qualification)

#### The ‘downgrading’ or rejection by secondary care of GP urgent referrals

Environmental context and resources reflecting the dynamics within the primary–secondary interface were also relevant to primary care cancer detection (physical Opportunity). GPs were influenced by awareness of resource constraints and the pressure on secondary care, and a desire not to add to an already strained system. Once a GP had made a decision to refer a patient with suspected cancer to secondary care, the referral process itself presented further challenges for GPs. They described this as fraught with hidden barriers and implicit social norms and expectations (social Opportunity). Most GPs had experienced a suspected cancer referral being returned to them by secondary care because it was considered an inappropriate referral. They talked frequently in interviews about this rejection or ‘downgrading’ of their urgent referrals. This experience gave rise to strong emotions of anger, frustration or exasperation, apprehension, and self-doubt (automatic Motivation). For many, it felt like a lack of trust and respect. It also hindered the careful processes of diagnosis and decision making that GPs described adopting (psychological Capability):

*‘In day-to-day work, I’m finding* [the process] *a little bit tricky … I can’t use my judgement to get the test quickly. But in my opinion, on certain occasions, it might be needed.’*

(Urban, high deprivation, 15– 25 years since first medical qualification)

Clearly GPs wanted their referrals to be accepted and their patients to be seen by secondary care. At the same time, they did not want to be perceived as ‘an outlier’ who over-(or under-) referred, as this had implications for their professional or personal identity and reputation (both social Opportunity and reflective Motivation), which, as described in the previous section, was important to them:

*‘A big part of me wants to refer everyone. Yeah, 3% risk, you’re more likely to catch that earlier diagnosis and get a curable disease. But at the same time, you don’t want to completely swamp the system so that no one’s getting it. It is very resource limited. It does affect my practice significantly.’*

(Urban, high deprivation, 15– 25 years since first medical qualification)

GPs stated they would have found it useful to receive feedback from secondary care on the reasons for the referral rejection. None of the GPs interviewed had had any feedback and described how this added another layer of difficulty in getting the referrals just right (physical Opportunity):

*‘If I see somebody with a chest problem that I suspect is lung cancer, I might do an urgent suspected cancer referral. Quite often, they’re downgraded by the hospital. So they look at what you send them and say, “No, we don’t think it’s that, we don’t have to see them in two weeks.” There’s quite a bit of that that goes on, which doesn’t really help matters.’*

(Urban, low deprivation, >30 years since first medical qualification)

#### Adaptations to ensure referral acceptance by secondary care

Faced with this situation, GPs described how they developed (often informal) ways to address these influences and ensure referral acceptance by secondary care. They employed a number of strategies to work around the possibility of referral rejection. GPs were critical of the need to adopt these approaches and tended to describe them in somewhat contemptuous terms. Through these approaches, the GPs sought to achieve a clear-cut, indisputable justification for referral to ensure that the patient would be seen by secondary care, by reducing or eliminating any apparent uncertainty about the validity of their referral.

One of the methods that GPs used to adapt to this challenging referral environment was increasing the work-up ‘until it was obvious’ that a referral was needed, for example, conducting additional investigations before referral to ‘tick the box’. The approaches used were often convoluted, including activities such as building, or maximising, relationships with relevant secondary care clinicians (social Opportunity), or explicitly working on the art of referral letter writing (psychological Capability), or conducting more investigations to build a picture that was more likely to be accepted by secondary care to avoid being seen as an ‘over-referrer’ (reflective Motivation):

*‘You learn what to put in a referral to make sure they get seen.’*

(Rural, low deprivation, <10 years since first medical qualification)

Taken together, these overlapping factors created tensions for the GPs in the cancer diagnosis and referral processes. Tensions emerged between the internal, individual considerations of GPs (their own judgement and how referrals feel unwelcome) and external, context-dependent pressures (NICE guidance and secondary care pressures). This creates a picture of considerable complexity and a range of sources of influence on their decision making.

### Practice culture and systems

Practice culture and systems can ameliorate tensions and complexity. The perspective of practice teams described in focus groups presented a picture of how some of this tension may be offset by increasing social and physical Opportunity via a supportive general practice culture and helpful practice-based systems (Box 1).

#### Practice culture

GPs were asked what helped them deal with this pressure and the challenges of making urgent suspected cancer referrals. They described enabling social influences and the importance of ‘practice culture’, with examples given of support, advice, or opinions from the team or a colleague (social Opportunity). After a difficult or discouraging attempt to refer a patient, for example, GPs reported that reassurance and validation from others was appreciated and valuable. In the focus groups, some practice teams described similar types of support and a collective sense that ‘we are all in it together’:

*‘I hope, and I think everyone would say, we feel like we work in a team.* [Group agrees]*. We’ve got different roles but just because I’m the doctor it doesn’t mean that what I say goes. I really rely on other people to feed back to me and for me to feed back to them. That’s how things work.’*

(Focus group GP, urban area, non-training practice)

Being ‘all in it together’ did seem to be important in general practices that described the most constructive culture. It was emphasised that this included all members of the practice team, not just GPs or clinical staff. Where this worked best, admin and management team members felt that they too could contribute to cancer detection, as part of a whole-team effort:

*‘I think on the phone you don’t see people do you, but at the desk you do. So you notice their weight, or the yellowness or, you know if you think there is something not right, I mention it to one of the doctors.’*

(Focus group receptionist, urban area, non-training practice)

#### Practice systems

In the focus groups, practice teams described the practice-wide systems they had in place to support the cancer diagnosis process (physical Opportunity). Participants’ accounts indicated that the two most effective systems that did, or could, support the process of primary care cancer referral were safety netting and communication (Box 1).

A wide range of safety-netting activities were described and discussed in the focus groups. These were mostly environmental resources, such as administrative or ‘back-office’ systems developed to make the process ‘failsafe’. Where they appeared to work best was within whole-practice systems, which all team members were aware of and contributed to:

*‘A forum to clarify policies and procedures … give scenarios to staff. There’s always things that you can do differently or better. It’s just a case of sharing ideas and agreeing on a way forward, really.’*

(Focus group practice manager, rural area, training practice)

Practice communication and relational dynamics were described in the focus groups as being incredibly important for the process of cancer detection and diagnosis. Open-door communication between staff was highly valued. Positive relationships among team members, and as a team, were explicitly recognised as a valuable, and hidden, aid to cancer diagnosis. Providing explicit opportunities for team members to attend gatherings, whether official meetings, chats over the kettle, or informal ‘huddles’, was widely felt to be an important facilitator of cancer detection and diagnosis:

*‘The on-call doctor system is excellent … if you’ve got any concerns, you know who’s on-call, you go and wait. When their patient comes out, you go in and speak to them.’*

(Focus group nurse, rural area, training practice)

## Discussion

### Summary

In this article we combine analysis of the granular detail of the beliefs, attitudes, and behaviours of GPs as they diagnose symptoms that could be cancer, with systems tensions and enablers at practice level. As an embedded qualitative study, it is the first that we are aware of to explore systems, cultures, and norms through practice-team focus groups, in addition to individual GP perspectives. Through the lens of behaviour change theory, we describe how the social and physical opportunities afforded by practice culture and systems can assist or inhibit GPs’ cancer referral behaviour. We describe a range of complex workarounds that GPs felt they needed to adopt to face, and sometimes resist, challenging external systems and pressures.

GP accounts presented complex motivational and decision-making processes involving a range of individual considerations based on formal guidance, individual judgement, and ‘gut feelings’. Guidelines are potentially double-edged, in some scenarios helping to reduce cognitive load and provide reassurance that a decision was made systematically, but with the potential to restrict personal autonomy in following ‘gut feeling’. This apparent disconnect between reflective and automatic motivational factors can add to difficulties in the clinical decision-making process. GPs especially valued and prioritised the patient-level factors they took into account in the diagnostic process. They described a tension between their tried-and-trusted (and dearly valued) internal skills and motivations for detecting and diagnosing cancer and the external systems they needed to engage with when referring patients to secondary care.

Focus group data revealed how some of this tension could be offset by a positive practice culture and helpful practice-based systems (open-door communication between staff members and a feeling that ‘we are all in it together’) via supportive social influences and relationships, alongside physical opportunities for reflection via group meetings or ‘huddles’. This highlights the importance of a ‘whole-practice’ approach in ameliorating these tensions.

### Strengths and limitations

The strengths of the study include the following. This embedded qualitative study benefitted greatly from being part of the wider mixed-methods intervention development phase for the Think Cancer! trial. Quantitative and literature review work packages ran in parallel to the qualitative study, feeding into each other for the intervention development. The qualitative study also benefitted from this multidisciplinary team, including PPI, clinicians, and non-clinicians and researchers with expertise in behaviour change theory. These different perspectives, experiences, and knowledge sets were notable strengths in the analysis interpretation.

Our qualitative approach, along with the use of COM-B and TDF, enabled us to obtain the rich insights presented here. We consider the combination of individual data from the interviews and team data from the focus groups to be a strength, both were needed to meet the study objectives. The participation of other, non-GP members of the practice team was valuable in facilitating comprehensive discussion around formal and informal general practice systems and norms. Such an approach is rarely employed.

The purposive sampling was conducted with considerable rigour, so samples for both parts of the study comprised a range of relevant characteristics. Topic guides based on the parallel work packages from the research programme (realist review and survey) were developed and embedded within the theoretical framework of the COM-B.

Limitations of the study include that the study was conducted in Wales, where health care and health policy differ in some ways from other parts of the UK and cancer policy has developed behind that of England. Data were collected before the COVID-19 pandemic. COVID-19 (and the pressures it imposed on both primary and secondary care) may have exacerbated the complexities and tensions we describe, and we recognise that the data may have been somewhat different had they been collected after (or during) the pandemic.

Interviews were conducted by telephone, which was advantageous for recruitment. Although it could be that some GPs were less inclined to disclose information over the telephone, the converse could also be true. Data were gathered from primary care teams, further research into the perspectives of patients and secondary care professionals would be beneficial.

### Comparison with existing literature

Our results concur with findings on GP reliance on ‘gut feeling’.^22^^–^25 However, we show how this can be met with frustration. For GPs, this creates tension between motivational factors, such as their autonomous professional judgement, and external or systemic requirements and considerations representing social norms and pressures.

This study further adds to research reporting on the range of issues that GPs consider when working towards a diagnosis of cancer.^23^^,^^24^^,^^26^^,^^27^ We add to this an understanding of how decision making at the GP level includes relational considerations and personal motivators such as confidence, fear of adverse consequences, self-respect, and integrity. Detecting cancer is therefore guided not only by external requirements, but also by motivational factors that GPs described as part of their cancer diagnostics process.

There is very little published research on the frustration created by rejected (or ‘downgraded’) cancer referrals. We describe GPs’ feelings of exasperation and their corresponding efforts to combat the system, such as learning how to polish a referral letter to ensure that patients receive prompt secondary care and to maintain professional integrity. Much has been published, over many decades, on the primary–secondary care interface and communication across the two systems.^28^^–^30 However, especially in systems that allow downgrades, referral communication between primary and secondary care remains an ongoing problem, and one that is under-researched. None of the GPs in the current study received feedback about why their referrals were downgraded; therefore, improved communication is a potential way to improve the interface between primary and secondary care.

Weller and colleagues explored the value of involving the whole primary care team in the process of cancer referral.^31^ Our focus groups supported this and provided examples. Descriptions of strong whole-team involvement included active roles taken by receptionists and administrative team members, and enabled the exploration of factors relating to social and physical opportunity within the general practice setting.

Although we are not the first to describe the range of influences on GPs in their diagnostic processes, less research relates to cancer specifically. There is literature on systemic factors external to primary care and their impact on GPs’ early referral of cancer symptoms.^32^^–^34 However, the evidence provided tends to be more generic rather than specific to cancer. The primary– secondary care interface literature further reminds us of long-reported challenges in joint working and information sharing,^32^^–^34 as well as the advantages of doing so. All of this work can provide important insights for the early diagnosis of cancer. However, the powerful contribution to understanding cancer diagnosis that this article makes is to combine in-depth qualitative findings on the perspectives of GPs with that of primary care teams. This analysis, in one study, of separate data from both individual GPs and their wider practice systems allowed a much wider and comprehensive understanding than other research. By doing so, we are able to highlight the tensions created and ways they can, and are, managed by primary care practices.

### Implications for research and practice

The results highlight a commonly overlooked problem of the dynamics shaping cancer detection and referral in primary care (and may be transferable beyond primary care). Further qualitative and quantitative research is needed to clarify what could help improve constraints on decision making at the GP level. In England, an urgent suspected cancer referral policy (for symptoms that GPs feel require secondary care investigation as soon as possible) has led to an increase in rapid referrals. However, the lack of diagnostic capacity and delays created by referral challenges, including rejections from secondary care, are likely to persist and need urgent attention. Action has begun with the NHS Long Term Plan commitment to diagnose more cancers at an earlier stage.^35^ However, recent political activity has delayed the publication of the 10-year cancer plan for England that was due in summer 2022.^36^

As primary care recovers from COVID-19 there will likely be permanent changes to the way practices operate in consultation and referral, and issues relating to backlogs in secondary care may persist for some time. The shift to remote consultation may have an impact on cancer detection in several ways, for example, patients may not wish to disclose certain symptoms over the telephone, the loss of the physical examination, limited capacity to use technology, and patient concerns regarding use of a doctor’s time during a crisis.^37^^,^^38^

To better understand the barriers to cancer diagnosis from the primary– secondary care interface, which in turn will provide insight into possible solutions for delays, the need for further research is urgent. Such research should include secondary care clinicians and patients whose experience with their own referral being ‘downgraded’ appears to have been entirely overlooked.

The dynamics we describe are often experienced by GPs as a tension between individual decision making (Capability and Motivation) and external considerations, such as pressures in secondary care (Opportunity). We also describe how this tension can be ameliorated by increasing Opportunity for positive practice cultures and systems, and reinforce the importance of whole-practice interventions.
